# Clinical Features of Reported Ethylene Glycol Exposures in the United States

**DOI:** 10.1371/journal.pone.0143044

**Published:** 2015-11-13

**Authors:** Meghan A. Jobson, Susan L. Hogan, Colin S. Maxwell, Yichun Hu, Gerald A. Hladik, Ronald J. Falk, Michael C. Beuhler, William F. Pendergraft

**Affiliations:** 1 University of North Carolina (UNC) School of Medicine, Chapel Hill, North Carolina, United States of America; 2 UNC Kidney Center, Division of Nephrology and Hypertension, Department of Medicine, Chapel Hill, North Carolina, United States of America; 3 Department of Biology, Duke Center for Systems Biology, Duke University, Durham, North Carolina, United States of America; 4 Carolinas Poison Center, Carolinas Medical Center, Charlotte, North Carolina, United States of America; Nottingham University, UNITED KINGDOM

## Abstract

**Background:**

Ethylene glycol is highly toxic and represents an important cause of poisonings worldwide. Toxicity can result in central nervous system dysfunction, cardiovascular compromise, elevated anion gap metabolic acidosis and acute kidney injury. Many states have passed laws requiring addition of the bittering agent, denatonium benzoate, to ethylene glycol solutions to reduce severity of exposures. The objectives of this study were to identify differences between unintentional and intentional exposures and to evaluate the utility of denatonium benzoate as a deterrent.

**Methods and Findings:**

Using the National Poison Data System, we performed a retrospective analysis of reported cases of ethylene glycol exposures from January 2006 to December 2013. Outcome classification was summed for intentionality and used as a basis for comparison of effect groups. There were 45,097 cases of ethylene glycol exposures resulting in 154 deaths. Individuals more likely to experience major effects or death were older, male, and presented with more severe symptoms requiring higher levels of care. Latitude and season did not correlate with increased exposures; however, there were more exposures in rural areas. Denatonium benzoate use appeared to have no effect on exposure severity or number.

**Conclusion:**

Deaths due to ethylene glycol exposure were uncommon; however, there were major clinical effects and more exposures in rural areas. Addition of denatonium benzoate was not associated with a reduction in exposures. Alternative means to deter ingestion are needed. These findings suggest the need to consider replacing ethylene glycol with alternative and less toxic agents.

## Introduction

Ethylene glycol is a synthetic, colorless, and odorless liquid that tastes sweet and is used primarily to produce plastic containers and polyester fibers and secondarily as the main component in engine antifreeze [1]. Despite its utility, ethylene glycol is highly toxic and represents an important and persistent cause of intentional and unintentional poisonings worldwide [1–5]. Toxicity occurs after enzymatic conversion of the parent alcohol to glycolic acid and oxalic acid, which both produce numerous clinical manifestations, including confusion, nausea, vomiting, central nervous system dysfunction, cardiovascular compromise, elevated anion gap metabolic acidosis, and acute kidney injury [6–9]. Treatment includes supportive care, inhibition of alcohol dehydrogenase with intravenous fomepizole [7,10–12], and renal replacement therapy when there is severe acidemia or end organ injury, including acute kidney injury or severe neurologic impairment [13]. Prior to approval of fomepizole by the FDA in 1997 for the treatment of ethylene glycol poisoning [10], ethanol, used to inhibit alcohol dehydrogenase, was a mainstay of therapy. Ethanol continues to be used worldwide, but serious adverse side effects, including sedation and hypoglycemia, limit its utility [14,15].

In an attempt to limit human and animal exposures to ethylene glycol, denatonium benzoate (commonly Bitrex^®^), the most bitter-tasting compound known to man, has been added to ethylene glycol in antifreeze to serve as a non-toxic deterrent in many countries, including the United Kingdom, Canada, and the United States [16,17]. The first law in the United States requiring its addition was passed in the state of Oregon in 1991 [18,19]. Sixteen other states followed suit, yet attempts to pass national legislation mandating addition of bittering agent to engine coolant and antifreeze have failed [20]. In 2012, the Consumer Specialty Products Association announced an agreement with the Humane Society Legislative Fund to voluntarily add this agent; however, previous limited state-specific analysis of the utility of denatonium benzoate showed no reduction in exposures [21–24].

We analyzed all cases of reported ethylene glycol exposures within the National Poison Data System (NPDS) between 2006 and 2013 to determine differences between intentional and unintentional exposures, and to evaluate the utility of denatonium benzoate as a deterrent.

## Methods

### Setting and Participants

The NPDS, maintained by the American Association of Poison Control Centers (AAPCC), houses case records from the United States Poison Control Network, which receives human and animal poison exposure reports in all 50 states, the District of Columbia, American Samoa, the Federated States of Micronesia, Guam, Puerto Rico, and the U.S. Virgin Islands (S1 Table) [3]. The NPDS is the largest poison-exposure surveillance database in the United States, and is routinely used by the Centers for Disease Control, Food and Drug Administration, Environmental Protection Agency, Consumer Product Safety Commission and Drug Enforcement Agency. In 2006, a real-time data-capture repository was established, which replaced and updated the previous repository. All data analyzed in this report were entered after the new system was put into place. The AAPCC reviewed and approved our request for data, and the Institutional Review Board of Carolinas HealthCare System (CHS) approved this study (CHS IRB File # 04-14-15EX). Data was anonymized and de-identified prior to analysis. All authors vouch for the accuracy and completeness of the data.

### Design Overview, Definitions, and Data Analysis

We identified all cases of ethylene glycol exposure in humans within the NPDS from January 1, 2006 to December 31, 2013 using the AAPCC codes for ethylene glycol (automotive products including antifreeze, generic code 051221) or ethylene glycol (excluding automotive, aircraft, or boat products, generic code 052160). All cases that were confirmed as non-exposures or multiple substance exposures and cases outside the United States were excluded from analysis (S2 Fig). Exposures were classified as intentional, unintentional, other, or unknown. Analysis included all exposure types unless otherwise noted.

We ascertained the following demographic characteristics from these data: age, weight in kilograms, date of ingestion, sex, routes of exposure, reason for exposure, clinical effects/complaints, medical interventions, and medical outcomes. Age was calculated using continuous reported variables except for unintentional ingestions by individuals less than 18 years old, which includes additional categorical classifications, including: ≤5 years, 6–12 years, teen, and “unknown child” that were included as discrete age categories. Weight values for children under two years old were validated to correct for unit errors (pounds instead of kilograms). This was performed by visualizing weight of all patients under two years old and comparing to 150% of the normal values by age referencing World Health Organization (WHO) growth charts [25]. Outliers were divided by 2.205 to convert pounds to kilograms (S1 Fig). Seasons were defined as spring (March 20^th^ to June 20^th^), summer (June 21^st^ to September 21^st^), fall (September 22^nd^ to December 20^th^), and winter (December 21^st^ to March 19^th^) as averaged from 2006 to 2013. The route of exposure was most commonly oral. Other routes of exposure include ocular, otic, inhalation/nasal, aspiration, parenteral, dermal, rectal, vaginal, and “unknown.” Cases with a non-oral route of exposure were excluded when measuring the effect of denatonium benzoate on outcomes. The reason for exposure is coded as “unintentional” (an unforeseen or unplanned event), “intentional” (the result of a purposeful action), “other” (malicious as well as contaminant/tampering), “adverse reaction,” or “unknown” (for distributions see S5 Table). Clinical effects are discrete data fields and coded as “related,” “unknown if related,” or “not related.” These reflect the assessment of the specialist in the attribution of that clinical effect to the toxicant in question. There are 131 discrete possible clinical effects associated with each case; “related” and “unknown if related” were grouped as present, and “not related” and “not coded/null” as not present.

We report four common clinical complaints (headache, nausea, vomiting and abdominal pain) and five specific and more serious effects commonly associated with ethylene glycol ingestion (seizures (single and multiple), seizures (status epilepticus), coma, anion gap acidosis, and kidney damage). The clinical effect “seizures (single/multiple)” was defined if either “seizures, single” or “seizures, multiple” was coded as present. The clinical effect “anion gap acidosis” was defined if either “elevated anion gap” or “metabolic acidosis” was coded as present. The clinical effect “kidney damage” was defined if any of the following clinical effects were coded as present: “creatinine elevated,” “oliguria,” “anuria,” and “renal failure.” Effects coded as “related” and “unknown if related” were summed for each variable. Previous work indicates that coding of effects can be variable and that it is appropriate to combine similar effects, especially when there is inherent redundancy in the definition of the clinical effect [26,27]. Data extracted from the reported therapies include two common interventions for critically ill patients (intubation and intravenous fluids) and three more specific interventions commonly recommended and administered to patients who have been exposed to ethylene glycol (ethanol, fomepizole, and renal replacement therapy). Therapies coded as “recommended and performed” and “performed” were summed for each variable.

Medical outcomes are reported by the NPDS using the following five mutually exclusive categories: no effect (no signs or symptoms due to exposure), minor effect (signs or symptoms were minimally bothersome and resolved rapidly, e.g., nausea), moderate effect (signs of symptoms were more pronounced, prolonged, or systemic in nature but did not require specific intervention, e.g., acid-base disturbance), major effect (signs or symptoms were life-threatening or required specific intervention, e.g., seizures), death (death resulting from exposure or a direct complication of the exposure), unrelated effect (exposure probably not responsible for the effects), and confirmed non-exposure (exposure later believed to not have occurred). Outcome classification was summed for intentionality (unintentional or intentional; other reasons were excluded for this analysis) and used as a basis for separation and comparison of effect groups. Outcomes were grouped into “minor or no effect,” “death or major effects,” or “moderate effect” for analysis. The contribution to fatality for death cases was requested; cases were excluded if the AAPCC reviewer did not feel that death was a result of ethylene glycol exposure. Outcomes were assessed overall, by intentionality, and by ICU use.

### Statistical Analyses

Categorical data are reported as summed frequency and percentages and compared using chi-square or Fisher’s exact tests as indicated. Continuous data are reported as mean and standard deviation and are compared using a two-tailed unpaired Student’s t-test or an analysis of variance (ANOVA) as indicated. In the case of a significant ANOVA, we applied the Bonferroni correction for multiple comparisons of differences between individual groups. We calculated Pearson correlation coefficients for state incidence proportion compared to population data. Statistical significance was determined using the Bonferroni correction for multiple comparisons (0.05/number of comparison variables) to determine the critical *p-*value of significance. Predictors of intentional ethylene glycol exposures and of death were examined using multivariable logistic regression models. Results are reported as odds ratios with 95% confidence intervals and *p*-values. Odds ratios represent the likelihood that subjects with particular characteristics intentionally ingested ethylene glycol (or died). Model discrimination was evaluated using the concordance statistic (C-statistic), with values ranging from 0 to 1, with those approaching 1 determining more discriminating models.

Data analysis was performed using Excel (Microsoft Excel for Mac 2011) and R (R Project for Statistical Computing, version 3.0.2, www.r-project.org) with plyr package (version 1.8) [28]. Statistical analyses were performed and graphs were generated using Prism 5.0 (Graph Pad for Mac OS X). To evaluate geographical trends, choropleth maps were generated using ArcGIS ArcMap (Esri Software, version 10.1) using Jenks natural breaks classification method and an Albers projection [29]. GIS files containing state and water feature boundary files were obtained from the National Historical Geographic Information System (NHGIS) and joined with data generated from the NPDS to generate maps [30]. Joined files were exported to Adobe Illustrator CS5 (Adobe) where maps were altered for presentation purposes. Logistic regression analyses were performed using SAS version 9.2 (SAS Institute).

## Results

### Characteristics of Individuals Exposed to Ethylene Glycol

We identified 45,097 individuals reported to have been exposed to ethylene glycol in the United States between 2006 and 2013 (S2 Fig depicts method for case inclusion and S3 Fig depicts number reported by year). Of these exposures, 7,070 (16%) were intentional, and 38,027 (84%) were unintentional (Table 1). Intentional and unintentional groups were significantly different (*p*<0.02) for all demographic and clinical variables shown except weight (adults), the season during which the ingestion occurred, and headache. Table 1 includes selected features most relevant to toxic alcohol ingestion, but does not include all possible classifications of signs and symptoms, therapies, or outcomes. Males (n = 33,943, 75%) comprised the majority of exposed individuals. Deaths (n = 154, <1%) made up a low percentage of reported clinical outcomes. The following were the most notable enriched characteristics of the intentional ingestion group: older age (mean 39.4 years versus 30.8, *p* < 0.001), female gender (30% versus 22%, *p* <0.001), kidney damage (20% versus <1%, *p* <0.001), fomepizole use (55% versus 3%, *p* <0.001), and need for renal replacement therapy (32% versus <1%, *p* <0.001). Outcomes were also worse for the intentional exposure group, with a higher frequency of major effects (22% versus <1%, *p* <0.001) and death (2% versus <1%, *p* <0.001).

**Table 1:** Baseline Characteristics of Patients with Ethylene Glycol Poisoning in the United States.

Characteristics	Total*		Intentional	Unintentional	*P*-value^†^
** Number of patients, N (%)**	**45097 (100)**		**7070 (16)**	**38027 (84)**	**—**
Age at intoxication	30.5 ± 17.23 9512		39.4 ± 15.0 6360	30.8 ± 17.1 31820	<0.001^‡^
Age ≥ 18 at intoxication	37.4 ± 14.4 32841		40.8 ± 14.2 6015	36.3 ± 14.2 25634	<0.001^‡^
Weight (kg), age ≥ 18	83.2 ± 25.3 7238		81.3 ± 21.9 1318	83.7 ± 26.1 5727	0.002^‡^
Female	11154 (25)		2094 (30)	8408 (22)	<0.001
Oral exposure	32992 (73)		6846 (97)	24746 (65)	<0.001
**Season at time of intoxication**					
Spring	11398 (25)		1890 (27)	9508 (25)	0.01
Summer	11467 (25)		1781 (25)	9686 (25.5)	—
Fall	11240 (25)		1749 (25)	9491 (25)	—
Winter	10992 (25)		1650 (23)	9342 (24.5)	—
**Reported signs and symptoms**					
Headache	1238 (3)		150 (2)	1088 (3)	<0.001
Nausea	2625 (6)		490 (7)	2135 (6)	<0.001
Vomiting	2437 (5)		872 (12)	1565 (4)	<0.001
Abdominal pain	897 (2)		308 (4)	589 (2)	<0.001
Seizure (single/multiple)	184 (<1)		172 (2)	12 (<1)	<0.001
Seizure (status)	22 (<1)		20 (<1)	2 (<1)	<0.001
Coma	716 (2)		679 (10)	37 (<1)	<0.001
Anion gap acidosis	2723 (6)		2491 (35)	232 (<1)	<0.001
Kidney damage	1516 (3)		1396 (20)	120 (<1)	<0.001
**Therapies**					
Intravenous fluids	4179 (10)		3243 (46)	936 (3)	<0.001
Ethanol	524 (1)		377 (5)	147 (<1)	<0.001
Fomepizole	4859 (11)		3858 (55)	1001 (3)	<0.001
Admitted to ICU	4193 (9)		3593 (51)	600 (2)	<0.001
Intubation	1210 (3)		1143 (16)	67 (<1)	<0.001
Renal replacement therapy	2456 (5)		2294 (32)	162 (<1)	<0.001
**Outcomes**					
Death	154 (<1)		144 (2)	10 (<1)	<0.001
Major effect	1672 (4)		1541 (22)	131 (<1)	<0.001
Moderate effect	3560 (8)		1654 (23)	1906 (5)	<0.001
Minor effect	7379 (16)		806 (11)	6573 (17)	<0.001

Plus-minus values are mean (SD) and number of patients reporting

*Total is intentional plus unintentional ingestions

^**†**^ *P-Value* compares intentional versus unintentional intoxication, Chi-square test unless otherwise noted, Bonferroni-corrected *p-value* for significance is <0.002 (0.05/28 comparison variables)

^‡^ *P-value* calculated using a Student’s unpaired two-tailed t-test

### Effects and Outcomes of Individuals Exposed to Ethylene Glycol

We compared characteristics of exposed individuals based on clinical severity of exposure and found significant differences for all comparisons. Overall, 21,895 poisoned individuals were followed to three categories of known outcome: minor or no effects (N = 16,155), moderate effects (N = 3714), and major effects or death (N = 2026, groups compared in Table 2). Individuals who died or sustained major effects were older (44 years) compared to those with no or only minor effects (29.8 years), and more often male (72% versus 77%) and exposed intentionally (93% versus 13%).

**Table 2:** Characteristics of Patients Who Experienced Effects Attributed to Ethylene Glycol Poisoning*.

Characteristics	Minor and no effects	Moderate effects	Death and major effects
**Number of patients, N (%)**	**16155**	**3714**	**2026**
Age at intoxication^†^	29.8 ± 17.5 14421	37.8 ± 15.2 3476	44.0 ± 15.0 1983
Age ≥ 18 at intoxication^†^	36.3 ± 14.3 11135	39.3 ± 14.2 3275	44.9 ± 14.3 1929
Weight (kg), age ≥ 18^‡^	82.0 ± 27.0 2994	80.9 ± 21.6 670	80.2 ± 21.9 424
Female^§^	3720 (23)	1003 (27)	576 (28)
Oral exposure^§^	11538 (71)	2383 (64)	1938 (96)
Unintentional^§^	14009 (87)	1910 (52)	144 (7)
Unintentional, age < 18^§^	3149 (20)	140 (4)	49 (2)
**Reported signs and symptoms**			
Headache	497 (3)	153 (4)	46 (2)
Nausea	1205 (8)	303 (8)	182 (9)
Vomiting	1041 (6)	382 (10)	344 (17)
Abdominal pain	396 (3)	153 (4)	95 (5)
Seizure (single/multiple)	4 (<1)	16 (<1)	192 (10)
Seizure (status)	0 (0)	2 (<1)	22 (1)
Coma	3 (<1)	123 (3)	664 (33)
Anion gap acidosis	43 (<1)	1234 (33)	1614 (80)
Kidney damage	14 (<1)	361 (10)	1246 (61)
**Therapies**			
Intravenous fluids	1268 (8)	1361 (37)	1452 (72)
Ethanol	176 (1)	156 (4)	151(8)
Fomepizole	1517 (9)	1624 (44)	1614 (80)
Admitted to ICU	918 (6)	1493 (40)	1712 (85)
Intubation	16 (<1)	261 (7)	1007 (50)
Renal replacement therapy	164 (1)	847 (23)	1534 (76)

Plus-minus values are mean (SD) with number of patients reporting. Bonferroni-corrected *p-*value for significance is <0.002 (0.05/19 comparison variables).

* Includes all classifications of intention, see methods

^**†**^ *P-*value calculated using Chi-square, *p*<0.001 for both age rows, compared by effects

^‡^ *P-*value calculated using Chi-square, *p* = 0.290 for weight, compared by effects

^§^ *P*-value calculated using ANOVA comparing three column effect variables is *p*<0.0001 for all row, variables shown

Corresponding to the definitions of a major effect from exposure, those in this group were more likely to suffer kidney damage (n = 1246, 61%), be admitted to the ICU (n = 1712, 85%), and undergo intubation (n = 1007, 50%) and renal replacement therapy (n = 1534, 76%). Analysis of therapeutic interventions between 2006 and 2013 revealed stable rates of intravenous fluid and fomepizole use, but a significant decrease in the requirement for renal replacement and ethanol therapies (S4A and S4B Fig). During this period, there was also a decrease in the number of deaths and major effects (S4C and S4D Fig).

**Fig 1:**
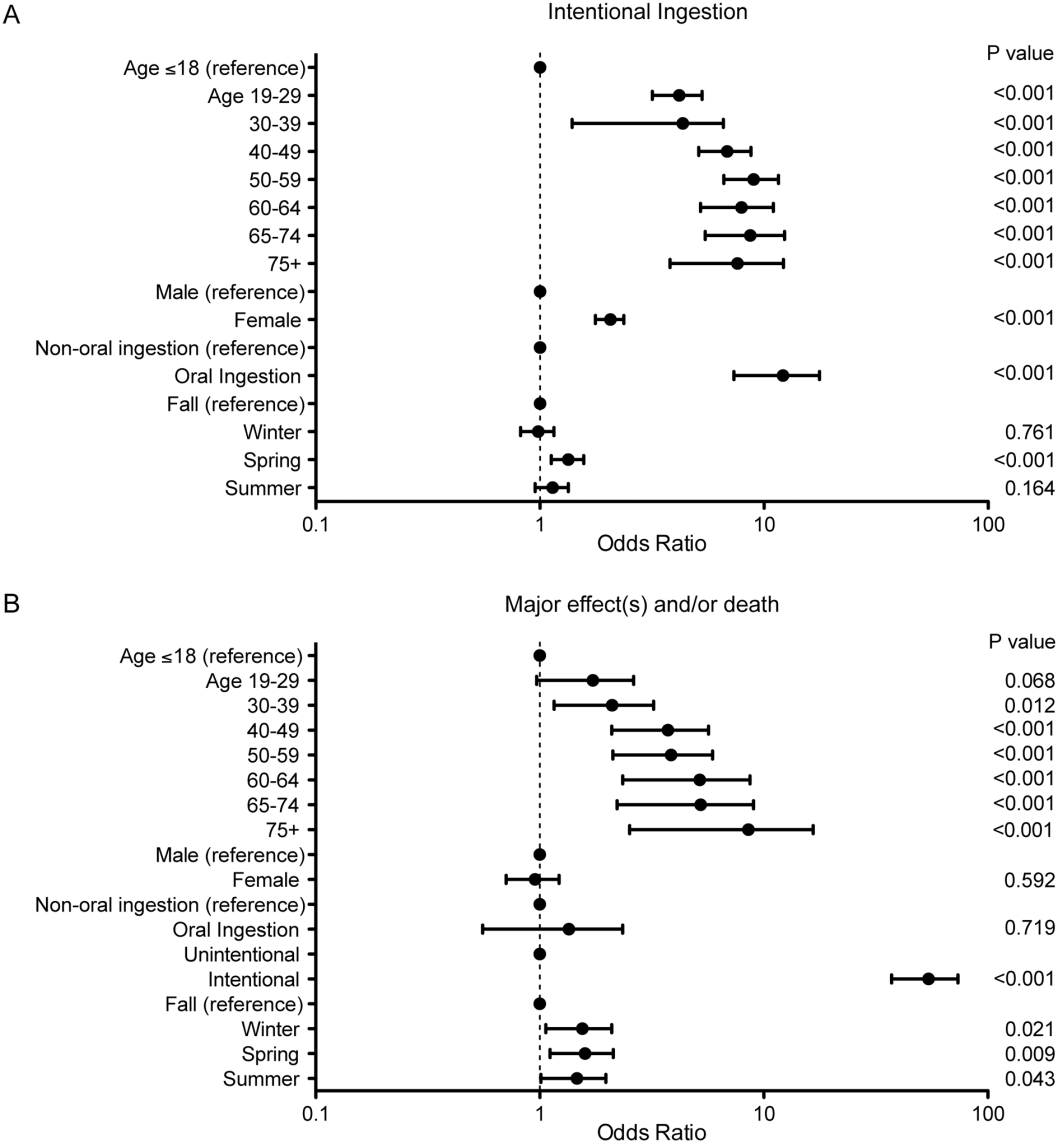
Logistic regression analysis of risk factors associated with ethylene glycol ingestion. Age is reported in years and each group is compared to age ≤ 18 years. Oral ingestion is compared to all known types of other exposures (unknowns excluded). Listed seasons are compared to Fall. Bonferroni-corrected *p*-value <0.003 (0.05/17 or 18 comparison variables) is considered significant. (A) Multivariate model of variables associated with intentional ingestion (C-statistic = 0.74). (B) Multivariate model of variables associated with major effect(s) and/or death (C-statistic = 0.92). See S4 Table for additional information.

Multivariable analysis revealed the following statistically significant predictors of intentional ethylene glycol exposure: age over 18 years, female gender, oral route of exposure, and spring compared to fall (Fig 1A, numerical data in S4 Table). Specifically with regards to gender, more men were exposed to ethylene glycol overall and more men intentionally ingested ethylene glycol; however, this analysis implies that women exposed to ethylene glycol are more likely to experience more serious effects. The risk of intentional ingestion for winter and summer fell between spring and fall and were not statistically distinct from either. Statistically significant predictors of major effect(s) and/or death were also identified: age 30 years and higher, spring compared to fall, and intentionality (Fig 1B). The risks of major effect(s) and/or death in winter and summer were similar to those in spring. These findings were identical when analyzing predictors of major effect(s) or death separately (data not shown). Model discrimination was strong in both models (intentional ingestion C-statistic = 0.74, major effect(s) and/or death C-statistic = 0.92).

### Geographic Trends of Ethylene Glycol Exposures

**Fig 2:**
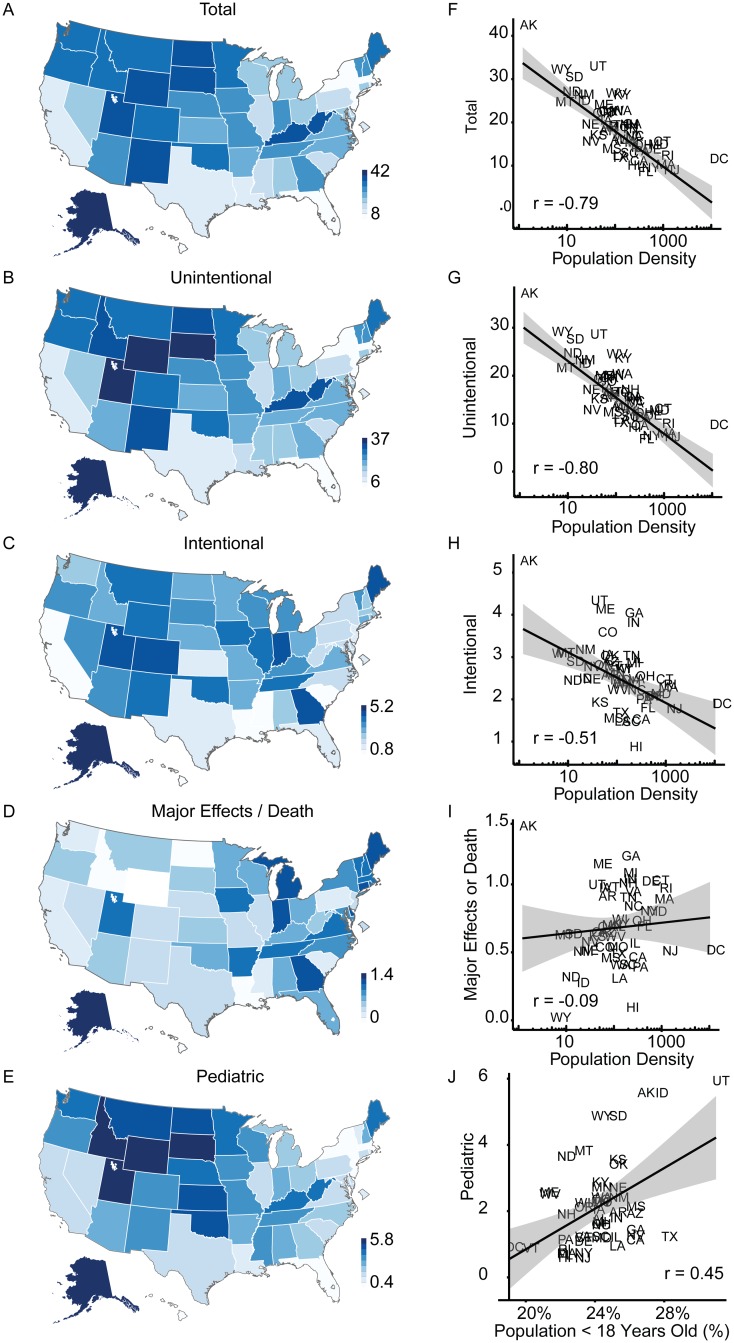
Incidence proportion of ethylene glycol exposures and population correlations. Choropleth maps show incidence proportion for ethylene glycol exposures for (A) all, (B) intentional, (C) unintentional, (D) major effects and death and (E) pediatric unintentional exposures (≤ 6 years old). Panels F-J show corresponding state incidence proportions correlated with population density (population per square mile) by state (F-H), panel J shows incidence of pediatric unintentional exposures correlated to percent of population of children under 18 years old by state. Incidence is per 100 000 humans, r = Pearson’s correlation coefficient.

Choropleth maps depicting frequency of ingestion by population density are shown in the United States and the District of Columbia and based on intentionality, major outcomes and deaths combined, and in the pediatric population (age <18) (Fig 2). We compared population density to the first four variables and the percent of population under the age of 18 for pediatric ingestions. There was a strong negative correlation between ethylene glycol exposures and population density by state for total, unintentional, and intentional ingestions. There was no correlation between population density and the incidence of major effects or death, or the frequency of unintentional ingestions in the pediatric population. We found no difference with regard to exposures by latitude. Alaska had the highest number of intentional and unintentional ingestions (S2 Table for rankings). Hawaii had the lowest number of intentional ingestions and Florida had the lowest number of unintentional ingestions.

### Effect of the Aversive Additive Denatonium Benzoate on Oral Ethylene Glycol Ingestion

**Fig 3:**
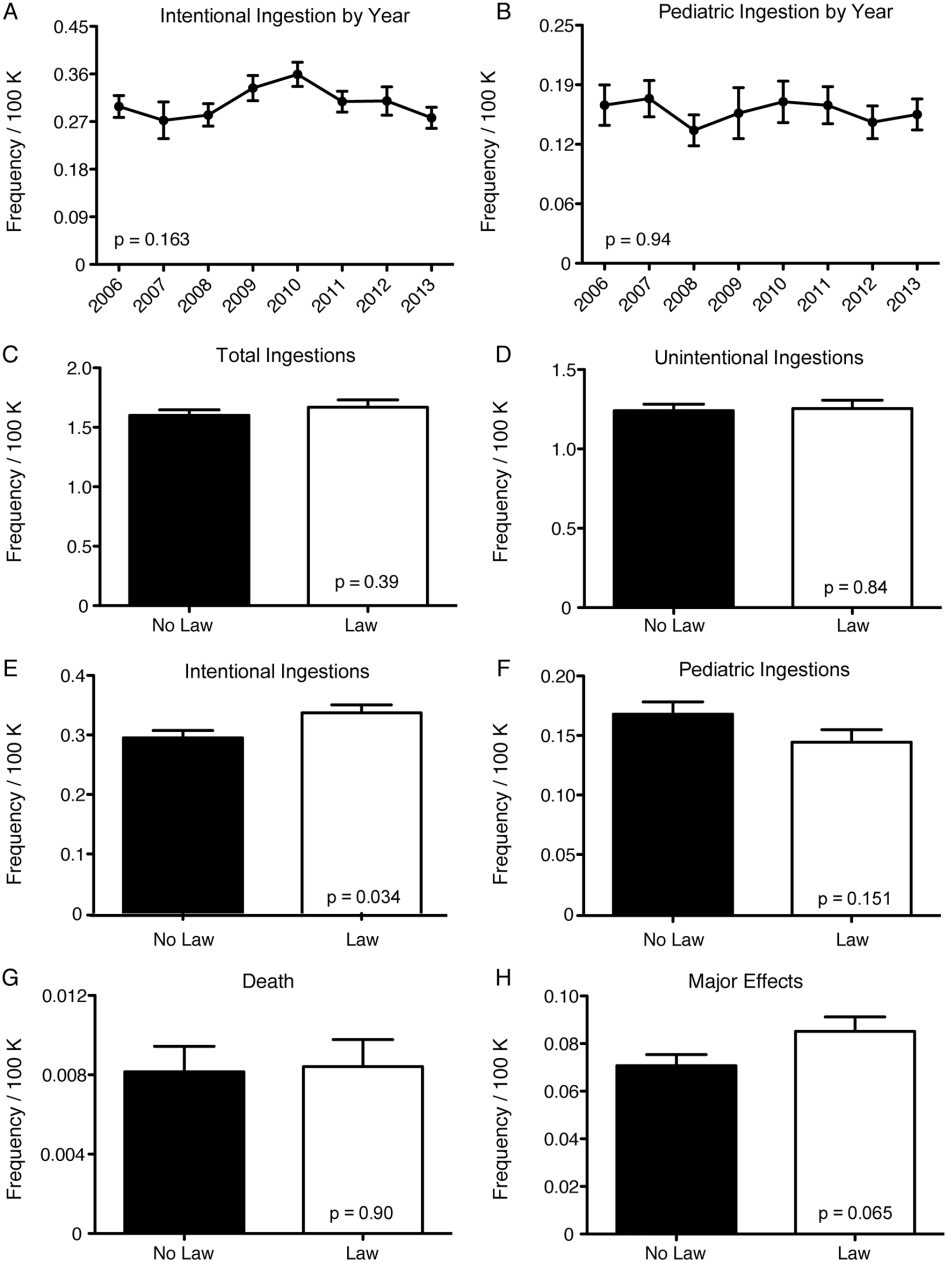
The effect of addition of bittering agent to ethylene glycol on frequency of oral ingestions in the United States and the District of Columbia. Panels A and B show incidence of intentional (> 11 years old) and pediatric unintentional oral ingestions (≤ 6 years old) per year for all states. In 2012, bitterant was added to commercially sold antifreeze in the United States and the District of Columbia. Panels C-F show no reduction in total, unintentional, intentional (> 11 years old), or pediatric unintentional oral ingestions (≤ 6 years old) of ethylene glycol in states that have added bitterant to ethylene glycol (n = 17) compared to those that have not (n = 34) from 2006–2013. Panels G and H show no reduction in death or major effects in states that have added bitterant compared to those that have not from 2006–2013. Y-axis represents frequency per 100,000 (100 K) humans for all panels; error is SEM; *p-*values calculated using analysis of variance for panels A and B.

Seventeen states now require addition of the bittering agent, denatonium benzoate, to ethylene glycol preparations as a means to deter individuals from ethylene glycol ingestion (S3 Table). Limiting our analysis to include only oral exposures, we found no significant difference when examining intentional and pediatric ingestions by year (Fig 3A and 3B). We examined the effect of this additive on oral ethylene glycol ingestions in states requiring addition of denatonium benzoate, as compared to states not requiring its addition, and found no significant difference in the total number of ingestions (*p* = 0.39), unintentional ingestions (*p* = 0.84), or pediatric ingestions (*p* = 0.151) (Fig 3C, 3D and 3F). In fact, there was a significant increase in the number of intentional ingestions in states with an enacted law (*p* = 0.034) (Fig 3E). Furthermore, there were no significant differences in deaths or major effects (Fig 3G and 3H).

## Discussion

This analysis of all reported ethylene glycol exposures within the United States over the past eight years reveals characteristics that differentiate individuals with intentional or unintentional exposures. Those who intentionally exposed themselves to ethylene glycol were more likely to be older, male, and to have more severe signs and symptoms at presentation that required more intensive therapy. Although deaths and major effects were uncommon and have been decreasing since 2006 [31], individuals who died and/or experienced major effects, regardless of intent, have more severe signs and symptoms and were more likely to require aggressive care. Multivariable analysis confirmed older age and female gender as predictors of intentionality as well as oral route of exposure; that is, for all individuals exposed to ethylene glycol, women were more likely to have been exposed intentionally, and those who ingested ethylene glycol orally were more likely to have done so intentionally. Deaths, major effects, and moderate effects were also more common in this group. Significant predictors of major effects and death included older age and intentionality. As previously reported, we confirmed that fomepizole use has supplanted ethanol as an antidote for ethylene glycol poisoning. This finding corroborates prior studies showing the benefit of fomepizole over ethanol [7,10].

Contrary to our expectations, cases of ethylene glycol exposure were not associated with northerly latitude or season. Another unexpected finding not shown previously was the association of cases of intentional and/or unintentional exposures with low population density; that is, states with low population density had more reported exposures per capita. This pattern has not been seen in other types of poisonings in the United States; however, there are low population density associations with other types of injuries. For example, a retrospective study recently reported that Americans in rural regions were more likely to die from unintentional firearm injuries than those in urban regions [32]. Additionally, suicide rates of rural youth are nearly double those of urban youth [33]. States with lower population density may have a greater need for vehicular use, which would suggest increased exposure to ethylene glycol. Those who live in areas of low population density may be more likely to service their own vehicle. This suggests that resources aimed at decreasing exposures should be targeted to rural areas.

Limited data exist regarding characteristics of individuals exposed to ethylene glycol and predictors of poor outcomes [34]. Analysis of California data revealed an association of more severe clinical signs with individuals exposed to ethylene glycol between 1999 and 2008 who died or had prolonged renal insufficiency. Improved outcomes were seen with earlier antidote administration [35]. The degrees of osmolal and anion gaps have also been associated with increased mortality [2]. More recently, analysis of NPDS data between 2000 and 2013 revealed that fomepizole use has essentially replaced ethanol as a treatment modality and that the use of renal replacement therapy is trending down [31]. Our analysis confirms these findings on a national level and provides additional insight into the link between intention and case outcome.

Most importantly, we found no change in the number of oral ingestions in states that have required addition of denatonium benzoate to ethylene glycol preparations. In fact, the number of intentional exposures has significantly increased. This suggests that the addition of an aversive agent to ethylene glycol may not reduce harm to those who are most affected—individuals who attempt to commit suicide by ethylene glycol ingestion—as was previously shown in two states [24]. One of the first commercial uses of denatonium benzoate was as an aversive bittering agent in a Danish pig farming community where it was applied to pigs’ tails to avoid cannibalization [36]. Studies have shown that small concentrations of denatonium benzoate were effective in reducing the volume of material swallowed by children [37]; subsequent studies have supported this finding [38–40]. It is unclear if addition of denatonium benzoate would prevent or lessen ethylene glycol poisoning, given that small volumes of ethylene glycol are toxic; however, denatonium benzoate may be effective in reducing severity rather than frequency of exposure by reducing the volume ingested, which requires future investigation. One could hypothesize that individuals who intentionally ingest ethylene glycol may be more likely to ingest large quantities regardless of bitterness given the intention to inflict self-harm. Single state studies reinforce our conclusions by demonstrating that addition of denatonium benzoate to ethylene glycol in Oregon and California did not affect rates of ethylene glycol poisoning in animals and humans [22–24]. These findings differ from those seen with legislation to prevent other types of fatalities. For example, more firearm laws are associated with fewer firearm-related fatalities in the United States [41].

One limitation of this study relates to NPDS data in general [42]. Case records reflect information provided when the public or healthcare professionals report an actual or potential exposure to a substance. Exposures do not necessarily represent a poisoning or overdose [43]. The AAPCC is not able to verify the accuracy of every report made to centers. There is also heterogeneity of coding practices across poison centers. Certain infrequent clinical effects are potentially coded less often than those with greater familiarity, but efforts were made to limit under-coding by combining related clinical effects to capture more severe effects. This confounder is likely similar across all centers and therefore, would not affect conclusions drawn here. Case outcomes can also be erroneously up-coded in severity, which could potentially result in greater reported severity of all cases, but comparison between intention and state incidence across time should not be affected. In addition, some fatalities could have been missed in individuals who died outside of a medical setting. Future efforts to identify such individuals are needed and could include analysis of state medical examiner death records. Additionally, prospective and longitudinal collection of clinical data from patients with ethylene glycol poisoning across major medical centers is warranted to validate and increase granularity of findings presented here.

Since its discovery by Charles-Adolphe Wurtz in 1856 [44], ethylene glycol has become a major commercial commodity. The ethylene glycol industry exceeds $21 billion in annual market value [45], and demand is increasing. In 2013, 86% of worldwide consumption of monoethylene glycol went into the production of polyethylene terephthalate (PET) (fibers, film and bottles), and 7.5% was used to produce antifreeze [46]. Alternative means to deter intentional and unintentional consumption are needed. For example, improved labeling and mandatory use of child-proof caps are effective in deterring consumption of toxic agents, at least in children [47]. Our findings suggest replacing ethylene glycol as a main component in antifreeze with alternative and less toxic agents with similar properties, such as propylene glycol or glycerol [48], as the morbidity associated with this product is significant, approved alternatives exist, and deterrents are, in our analysis, ineffective. This study is the first to provide an in-depth analysis of demographic, clinical, and therapeutic data related to trends in ethylene glycol exposures in the United States, and suggests the need to identify and implement more effective preventative strategies to reduce ethylene glycol poisoning.

## Supporting Information

S1 Fig Determination of weight of all ethylene glycol-exposed individuals under two years of age. Weight values for children under two years of age were validated to correct for unit errors (pounds instead of kilograms) that occurred during data entry. This was performed by visualizing weight of all patients under two years of age and comparing the values to 150% of the normal values by age (WHO growth charts for reference). Panel A shows two lines fit to the data using growth chart data. Panel B shows the data after the outliers were divided by 2.205 to convert pounds to kilograms with the assumption that outliers were incorrectly entered in pounds instead of kilograms.
(TIF)

S2 Fig Flow chart of study subject selection. (PDF)

S3 Fig Incidents of ethylene glycol exposures from 2006–2013. Number of intentional, unintentional and other (defined as “malicious,” “contamination,” “tampering,” “adverse reaction,” and “unknown”) exposures in the United States and District of Columbia plotted by year. There were no statistically significant differences across the inclusion period.
(PDF)

S4 Fig Trends in therapies and outcomes after ethylene glycol exposure. Panels A and B show trends in use of therapies. Use of intravenous fluid and fomepizole have remained constant while use of renal replacement therapy and ethanol has declined. Panels C and D show trends in deaths and major effects. X-axis is years and Y-axis is number of times therapy or outcome was reported in year x per cases reported in year x.
(TIF)

S1 Table Poison Control Centers within the United States Poison Control Network. (PDF)

S2 Table Rank of Top 20 States Based on Number of Intentional or Unintentional Ingestions during the Study Period. (PDF)

S3 Table Years that States Required Addition of Denatonium Benzoate to Ethylene Glycol Based Antifreeze Formulations. (PDF)

S4 Table Statistics from Logistic Regression Analysis of Risk Factors Associated with Ethylene Glycol Ingestion (Fig 1). (DOCX)

S5 Table Reported Reason of Exposure to Ethylene Glycol. (DOCX)

## References

[1] Cavender FL, Sowinski EJ. Patty’s industrial hygiene and toxicology. 4 ed. Clayton G, Clayton F, editors. New York: John Wiley & Sons, Inc; 1994 pp. 4645–4657.

[2] Coulter CV, Farquhar SE, McSherry CM, Isbister GK, Duffull SB. Methanol and ethylene glycol acute poisonings—predictors of mortality. Clinical Toxicology. 2011;49: 900–906. 10.3109/15563650.2011.630320 22091788

[3] Bronstein AC, Spyker DA, Cantilena LR Jr, Rumack BH, Dart RC. 2011 Annual Report of the American Association of Poison Control Centers’ National Poison Data System (NPDS): 29th Annual Report. Clinical Toxicology. 2012;50: 911–1164. 10.3109/15563650.2012.746424 23272763

[4] Wax PM. Elixirs, diluents, and the passage of the 1938 Federal Food, Drug and Cosmetic Act. Ann Intern Med. 1995;122: 456–461. 7856995 10.7326/0003-4819-122-6-199503150-00009

[5] Alkahtani S, Sammons H, Choonara I. Epidemics of acute renal failure in children (diethylene glycol toxicity). Arch Dis Child. 2010;95: 1062–1064. 10.1136/adc.2010.183392 21062849

[6] Haggerty RJ. Toxic hazards. Deaths from permanent antifreeze ingestion. N Engl J Med. 1959;261: 1296–1297. 10.1056/NEJM195912172612514 13855883

[7] Brent J. Current management of ethylene glycol poisoning. Drugs. 2001;61: 979–988. 11434452 10.2165/00003495-200161070-00006

[8] Berman LB, Schreiner GE, Feys J. The nephrotoxic lesion of ethylene glycol. Ann Intern Med. 1957;46: 611–619. 13403542 10.7326/0003-4819-46-3-611

[9] Kahn HS, Brotchner RJ. A recovery from ethylene glycol (anti-freeze) intoxication; a case of survival and two fatalities from ethylene glycol including autopsy findings. Ann Intern Med. 1950;32: 284–294. 15403194 10.7326/0003-4819-32-2-284

[10] Brent J. Fomepizole for ethylene glycol and methanol poisoning. N Engl J Med. 2009;360: 2216–2223. 10.1056/NEJMct0806112 19458366

[11] Brent J, McMartin K, Phillips S, Burkhart KK, Donovan JW, Wells M, et al Fomepizole for the treatment of ethylene glycol poisoning. Methylpyrazole for Toxic Alcohols Study Group. N Engl J Med. 1999;340: 832–838. 10.1056/NEJM199903183401102 10080845

[12] Baud FJ, Galliot M, Astier A, Bien DV, Garnier R, Likforman J, et al Treatment of ethylene glycol poisoning with intravenous 4-methylpyrazole. N Engl J Med. 1988;319: 97–100. 10.1056/NEJM198807143190206 3380132

[13] Caravati EM, Erdman AR, Christianson G, Manoguerra AS, Booze LL, Woolf AD, et al Ethylene glycol exposure: an evidence-based consensus guideline for out-of-hospital management. Clinical toxicology (Philadelphia, Pa.). 2005 pp. 327–345. 10.1080/07313820500184971 16235508

[14] Peterson CD, Collins AJ, Himes JM, Bullock ML, Keane WF. Ethylene glycol poisoning: pharmacokinetics during therapy with ethanol and hemodialysis. N Engl J Med. 1981;304: 21–23. 10.1056/NEJM198101013040105 7432434

[15] Wacker WEC. Treatment of ethylene glycol poisoning with ethyl alcohol. JAMA. 1965;194: 1231 10.1001/jama.1965.03090240065018 5897748

[16] Guinness World Records 2013. Guinness World Records; 2012.

[17] British Columbia Regulation 142/2009. Antifreeze regulation in the Environmental Management Act. British Columbia, 2009.

[18] Oregon Revised Statute 431.880. Aversive agent required. State of Orgeon, 1991.

[19] Oregon Revised Statute 431.885. Toxic household products required to comply with aversive agent requirement; exemptions. State of Orgeon, 1991.

[20] U.S. Senate. 109th Congress, First session S. 1110, the engine coolant and antifreeze bittering agent act of 2005: hearing before the Subcommittee on Consumer Affairs, Product Safety. Washington: Government Printing Office, 2005.

[21] Consumer Safety Product Association. Making antifreeze and engine coolant unpalatable to humans and animals. Available: http://www.cspa.org/advocacy/our-issues/129.html, Accessed June 13, 2014.

[22] Mullins ME, Zane Horowitz B. Was it necessary to add Bitrex (denatonium benzoate) to automotive products? Vet Hum Toxicol. 2004;46: 150–152. 15171494

[23] White NC, Litovitz T, Benson BE, Horowitz BZ, Marr-Lyon L, White MK. The Impact of Bittering Agents on Pediatric Ingestions of Antifreeze. Clin Pediatr (Phila). 2009;48: 913–921. 10.1177/0009922809339522 19571333

[24] White NC, Litovitz T, White MK, Watson WA, Benson BE, Horowitz BZ, et al The impact of bittering agents on suicidal ingestions of antifreeze. Clinical Toxicology. 2008;46: 507–514. 10.1080/15563650802119700 18584362

[25] WHO child growth standards: growth velocity based on weight, length and head circumference methods and development. World Health Organization; 2009.

[26] Beuhler MC, Wittler MA, Ford M, Dulaney AR. A controlled evaluation of case clinical effect coding by poison center specialists for detection of WMD scenarios. Clinical Toxicology. 2011;49: 684–690. 10.3109/15563650.2011.598530 21819293

[27] Sasser H, Nussbaum M, Beuhler M, Ford M. Classification tree methods for development of decision rules for botulism and cyanide poisoning. J Med Toxicol. 2008;4: 77–83. 18570166 10.1007/BF03160959 PMC3550141

[28] Wickham H. The split-apply-combine strategy for data analysis. Journal of Statistical Software. 2011;40: 1–29.

[29] Synder JP. Map projections: a working manual. Washington, DC: U.S. Department of the Interior, U.S. Geological Survey; 2011 Nov pp. 1–397. Report No.: 1395.

[30] Minnesota Population Center. National Historical Geographic Information System: Version 2.0. Minneapolis, MN: University of Minnesota 2011 Available: http://www.nhgis.org.

[31] Ghannoum M, Hoffman RS, Mowry JB, Lavergne V. Trends in toxic alcohol exposures in the United States from 2000 to 2013: a focus on the use of antidotes and extracorporeal treatments. Semin Dial. 2014;27: 1–7. 24712848 10.1111/sdi.12237

[32] Carr BG, Nance ML, Branas CC, Wolff CS, Kallan MJ, Myers SR, et al Unintentional firearm death across the urban-rural landscape in the United States. J Trauma Acute Care Surg. 2012;73: 1006–1010. 22976424 10.1097/TA.0b013e318265d10a

[33] Fontanella CA, Hiance-Steelesmith DL, Phillips GS, Bridge JA, Lester N, Sweeney HA, et al Widening Rural-Urban Disparities in Youth Suicides, United States, 1996–2010. JAMA Pediatr. 2015 10.1001/jamapediatrics.2014.3561 PMC4551430 25751611

[34] Porter WH, Rutter PW, Bush BA, Pappas AA, Dunnington JE. Ethylene glycol toxicity: the role of serum glycolic acid in hemodialysis. J Toxicol Clin Toxicol. 2001;39: 607–615. 11762669 10.1081/clt-100108493

[35] Lung DD, Kearney TE, Brasiel JA, Olson KR. Predictors of death and prolonged renal insufficiency in ethylene glycol poisoning. J Intensive Care Med. 2013; 10.1177/0885066613516594 24371252

[36] Bitrex history. Available: http://www.bitrex.com/en-us/about-bitrex/history. Accessed 21 May 2014.

[37] Berning CK, Griffin JL, Wild J. Research on the effectiveness of denatonium benzoate as a deterrent to liquid detergent ingestion by children. Fundamental and Applied Toxicology. 1982;2: 44–48. 10.1016/S0272-0590(82)80063-8 7185601

[38] Klein-Schwartz W. Denatonium benzoate: review of efficacy and safety. Vet Hum Toxicol. 1991;33: 545–547. 1808826

[39] Sibert JR, Frude N. Bittering agents in the prevention of accidental poisoning: children's reactions to denatonium benzoate (Bitrex). Arch Emerg Med. 1991;8: 1–7. 1854387 10.1136/emj.8.1.1 PMC1285726

[40] Jackson MH, Payne HA. Bittering agents: their potential application in reducing ingestions of engine coolants and windshield wash. Vet Hum Toxicol. 1995;37: 323–326. 8540219

[41] Fleegler EW, Lee LK, Monuteaux MC, Hemenway D, Mannix R. Firearm legislation and firearm-related fatalities in the United States. JAMA Intern Med. 2013;173: 732–740. 10.1001/jamainternmed.2013.1286 23467753

[42] Hoffman RS. Understanding the limitations of retrospective analyses of poison center data. Clin Toxicol (Phila). 2007;45: 943–945. 10.1080/15563650701233370 18163236

[43] Lévy A, Bailey B, Letarte A, Dupuis C, Lefebvre M. Unproven ingestion: an unrecognized bias in toxicological case series. Clin Toxicol (Phila). 2007;45: 946–949. 10.1080/15563650701197096 18163237

[44] Wurtz C-A. Sur le glycol ou alcohol diatomique. Comptes Rendus. 1856;43: 199–204.

[45] Prospects look good for global ethylene oxide, ethylene glycol markets. In: Processing Magazine: Solutions for the Process Industry. 8 Aug 2013.

[46] Chinn H, Kumamoto T. Mono-, Di-, and Triethylene Glycols. IHS Report; 2013 Nov. Report No.: http://chemical.ihs.com/CEH/Public/Reports/652.4000.

[47] Sibert JR, Craft AW, Jackson RH. Child-resistant packaging and accidental child poisoning. Lancet. 1977;35: 289–290. 10.1016/s0140-6736(77)90966-7 69891

[48] Hudgens RD, Hercamp RD, Francis J, Nyman DA, Bartoli Y. An evaluation of glycerin (glycerol) as a heavy duty engine antifreeze/coolant base. Warrendale, PA: SAE International; 2007 10 pp. 2007–01–4000. 10.4271/2007-01-4000

