# Supplementary figures and images for: Clinical Features of Reported Ethylene Glycol Exposures in the United States
Source: PLoS One. 2015 Nov 13;10(11):e0143044. doi: 10.1371/journal.pone.0143044 (PMC4643878; doi:10.1371/journal.pone.0143044)

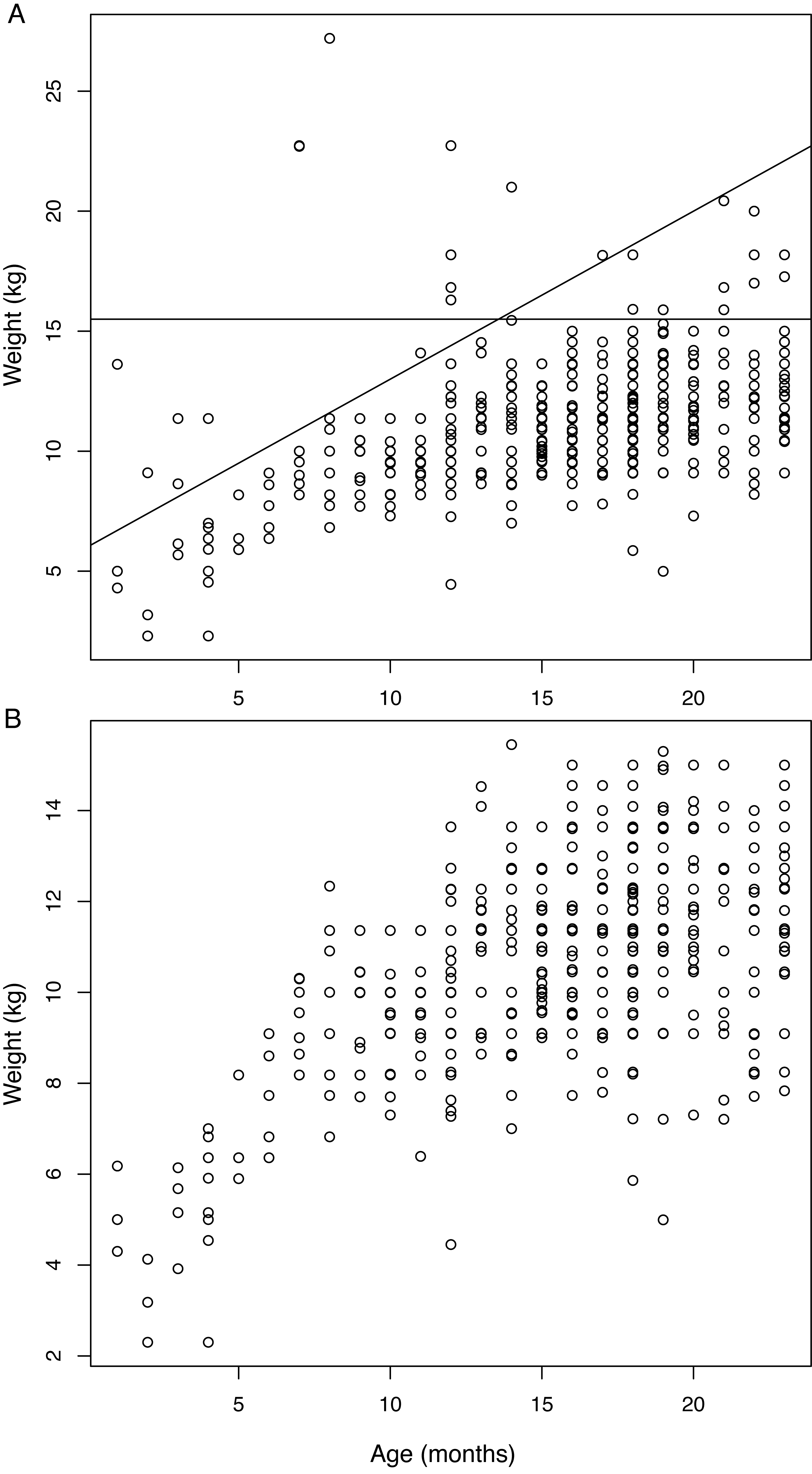

Supplement: S1 Fig — Weight values for children under two years of age were validated to correct for unit errors (pounds instead of kilograms) that occurred during data entry. This was performed by visualizing weight of all patients under two years of age and comparing the values to 150% of the normal values by age (WHO growth charts for reference). Panel A shows two lines fit to the data using growth chart data. Panel B shows the data after the outliers were divided by 2.205 to convert pounds to kilograms with the assumption that outliers were incorrectly entered in pounds instead of kilograms. (TIF) [file pone.0143044.s001.tif]

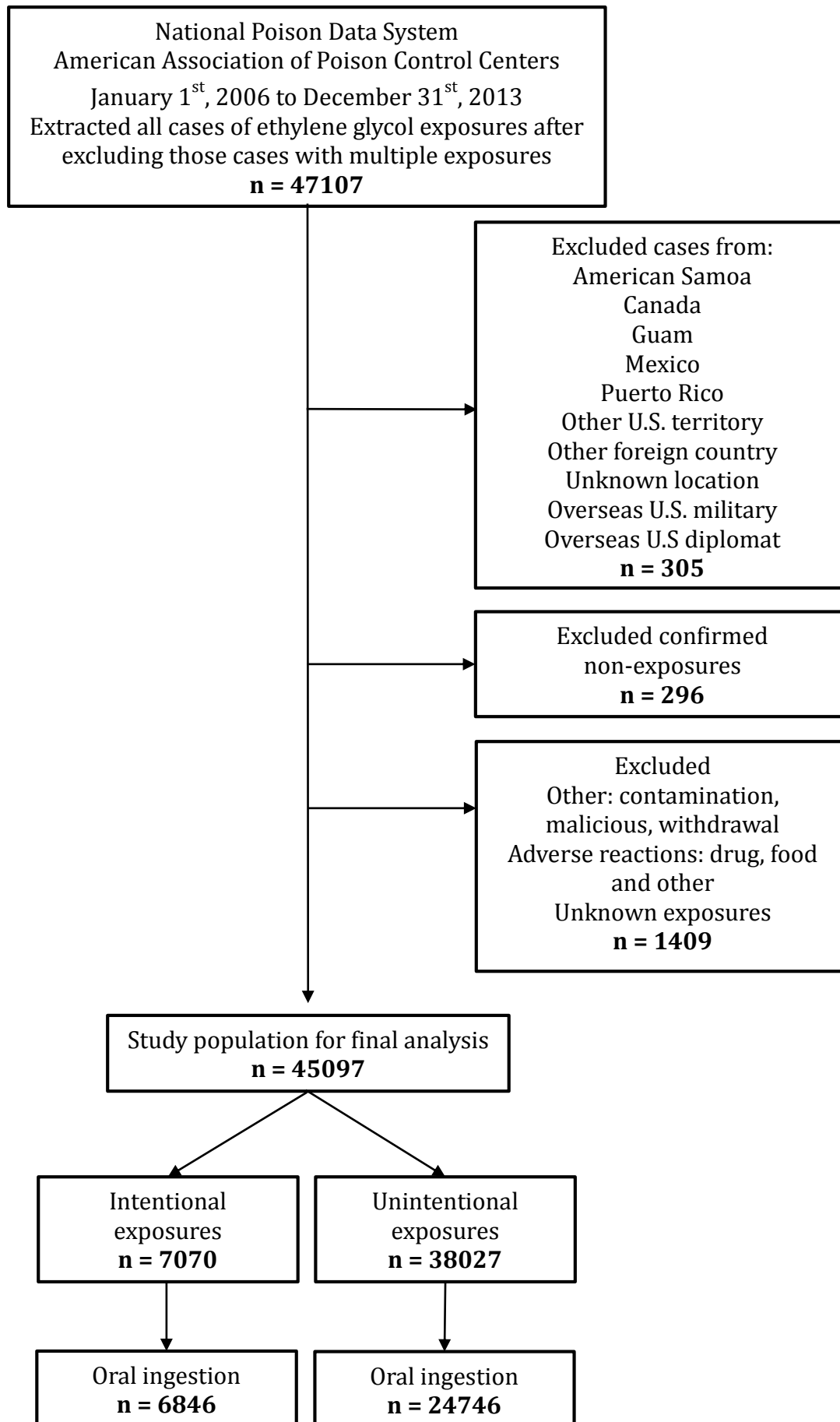

Supplement: S2 Fig — (PDF) [file pone.0143044.s002.pdf]

Incidents Per Year by Intention

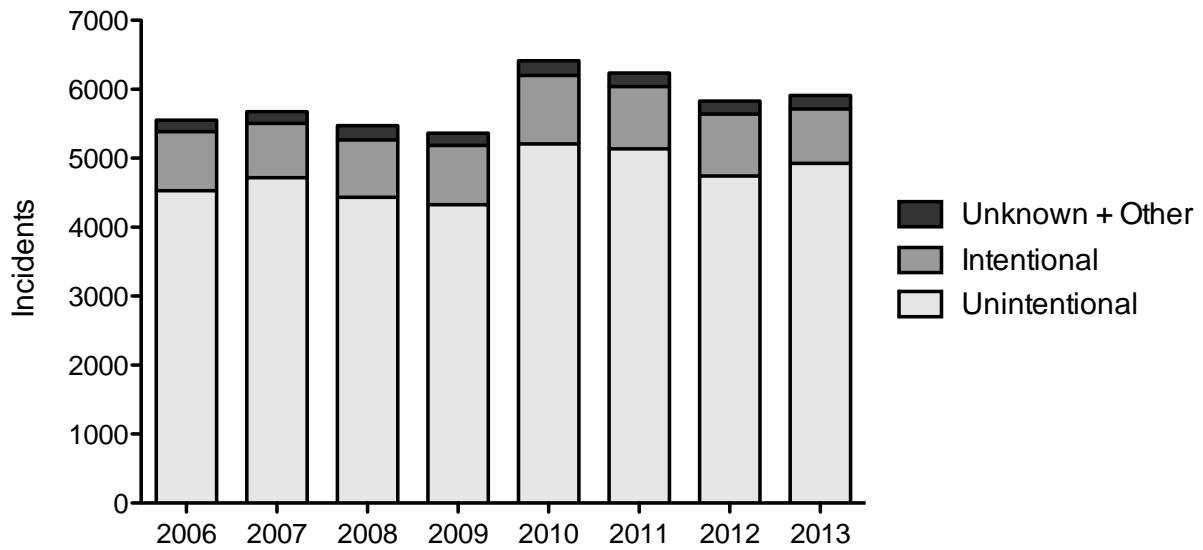

Supplement: S3 Fig — Number of intentional, unintentional and other (defined as “malicious,” “contamination,” “tampering,” “adverse reaction,” and “unknown”) exposures in the United States and District of Columbia plotted by year. There were no statistically significant differences across the inclusion period. (PDF) [file pone.0143044.s003.pdf]

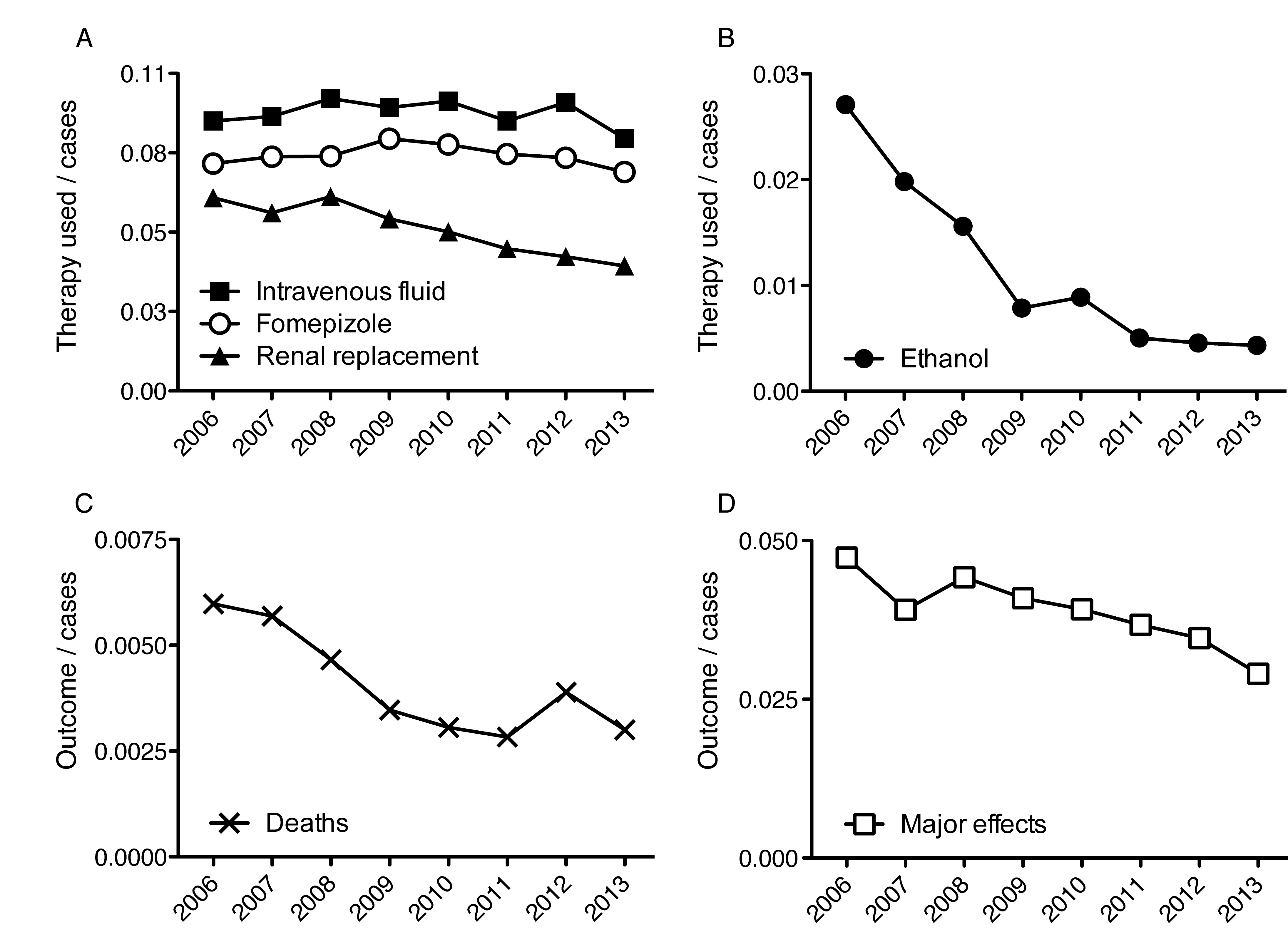

Supplement: S4 Fig — Panels A and B show trends in use of therapies. Use of intravenous fluid and fomepizole have remained constant while use of renal replacement therapy and ethanol has declined. Panels C and D show trends in deaths and major effects. X-axis is years and Y-axis is number of times therapy or outcome was reported in year x per cases reported in year x. (TIF) [file pone.0143044.s004.tif]
