# Supplementary material for: Clinical Features of Reported Ethylene Glycol Exposures in the United States
Source: PLoS One. 2015 Nov 13;10(11):e0143044. doi: 10.1371/journal.pone.0143044 (PMC4643878; doi:10.1371/journal.pone.0143044)
Supplement: S1 Table — (PDF) [file pone.0143044.s005.pdf]

S1 Table. Poison Control Centers within the United States Poison Control Network

Local Poison Control Center Name*	State, Country or Province	Included in Study
Arizona Poison & Drug and Information Center	Arizona	YES
Arkansas Poison and Drug Information Center	Arkansas	YES
Banner Good Samaritan Poison and Drug Information Center	Arizona	YES
British Columbia Drug & Poison Information Centre	British Columbia	NO
Blue Ridge Poison Center	Virginia	YES
California Poison Control System Central Office	California	YES
California Poison Control System - Fresno/Madera Division	California	YES
California Poison Control System - Sacramento Division	California	YES
California Poison Control System - San Diego Division	California	YES
California Poison Control System - San Francisco Division	California	YES
Carolinas Poison Center	North Carolina	YES
Central Ohio Poison Center	Ohio	YES
Central Texas Poison Center	Texas	YES
Cincinnati Drug and Poison Information Center	Ohio	YES
Connecticut Poison Center	Connecticut	YES
Department of Energy - Oak Ridge Operations Center (DOE-OROC)	Tennessee	YES
Florida Poison Information Center - Miami	Florida	YES
Florida Poison Information Center - Tampa	Florida	YES
Florida/USVI Poison Information Center - Jacksonville	Florida	YES
Georgia Poison Center	Georgia	YES
Hennepin Regional Poison Center	Minnesota	YES
Illinois Poison Center	Illinois	YES
Indiana Poison Center	Indiana	YES
Iowa Poison Control Center	Iowa	YES
IWK Regional Poison Centre - IWK Health Centre	Nova Scotia	NO
Kentucky Regional Poison Center	Kentucky	YES
Louisiana Poison Center	Louisiana	YES
Manitoba Poison Control Centre	Manitoba	NO
Maryland Poison Center	Maryland	YES
Mississippi Poison Control Center	Mississippi	YES
Missouri Poison Center at SSM Cardinal Glennon Children's Medical Center	Missouri	YES
National Capital Poison Center	District of Columbia	YES
National Capital Poison Center (Arlington, Fairfax, Fauquier, Loudoun, Prince William, and Stafford Counties, Alexandria, Fairfax, Falls Church, Manassas, Manassas Park Cities)	District of Columbia	YES
National Capital Poison Center	Also includes Virginia	YES
National Capital Poison Center (Montgomery and Prince George's Counties)	District of Columbia	YES
National Capital Poison Center	Also includes Maryland	YES
Nebraska Regional Poison Center	Nebraska	YES

Nebraska Regional Poison Center	Also includes Wyoming, Idaho, and American Samoa	YES
New Jersey Poison Information and Education System	New Jersey	YES
New Mexico Poison and Drug Information Center	New Mexico	YES
New York City Poison Control Center	New York	YES
Northern New England Poison Center	Maine	YES
Northern Ohio Poison Center	Ohio	YES
North Texas Poison Center	Texas	YES
Oklahoma Poison Control Center	Oklahoma	YES
Ontario Poison Centre	Ontario	NO
Oregon Poison Center	Oregon	YES
Oregon Poison Center	Also includes Alaska and Guam	YES
Palmetto Poison Center	South Carolina	YES
Pittsburgh Poison Center	Pennsylvania	YES
Poison and Drug Information Services of Alberta	Alberta	NO
Puerto Rico Poison Control Center, Inc.	Puerto Rico	NO
Quebec Poison Control Centre	Quebec	NO
Regional Center for Poison Control and Prevention Serving Massachusetts and Rhode Island	Massachusetts	YES
Regional Center for Poison Control and Prevention Serving Massachusetts and Rhode Island	Also includes Rhode Island	YES
Regional Poison Control Center Children's Hospital of Alabama	Alabama	YES
Rocky Mountain Poison & Drug Center	Colorado	YES
Rocky Mountain Poison & Drug Center	Also includes Hawaii, Montana, and Nevada	YES
Southeast Texas Poison Center	Texas	YES
South Texas Poison Center	Texas	YES
Sykes Telehealth Poison Information Centre	New Brunswick	NO
Tennessee Poison Center	Tennessee	YES
Texas Panhandle Poison Center	Texas	YES
The Poison Control Center of Pennsylvania	Pennsylvania	YES
The Poison Control Center of Pennsylvania	Also includes Delaware	YES
The University of Kansas Hospital Poison Control Center	Kansas	YES
Upstate New York Poison Center	New York	YES
Utah Poison Control Center	Utah	YES
VHS Children's Hospital of Michigan Regional Poison Control Center	Michigan	YES
Virginia Poison Center	Virginia	YES
Washington Poison Center	Washington	YES
West Texas Regional Poison Center	Texas	YES
West Virginia Poison Center	West Virginia	YES
Wisconsin Poison Center	Wisconsin	YES

* data obtained from http://www.aapcc.org/centers
