# Supplementary material for: Clinical Features of Reported Ethylene Glycol Exposures in the United States
Source: PLoS One. 2015 Nov 13;10(11):e0143044. doi: 10.1371/journal.pone.0143044 (PMC4643878; doi:10.1371/journal.pone.0143044)
Supplement: S2 Table — (PDF) [file pone.0143044.s006.pdf]

S2 Table. Rank of Top 20 States Based on Number of Intentional or Unintentional Ingestions during the Study Period.

Intentional	Unintentional
Alaska	Alaska
Utah*	Wyoming
Maine*	Utah*
Georgia	South Dakota
Indiana	North Dakota
Colorado	West Virginia*
Wyoming	Kentucky
New Mexico*	New Mexico*
Montana	Idaho
Iowa	Montana
Tennessee*	Washington*
Oklahoma	Minnesota
Illinois*	Maine*
Michigan	Oklahoma
Oregon*	Oregon*
Arizona*	Colorado
South Dakota	Iowa
Vermont*	New Hampshire
Nevada	Nebraska
Minnesota	Vermont*
*State with law requiring bittering agent in ethylene glycol-based antifreeze preparations	
