# Supplementary material for: Clinical Features of Reported Ethylene Glycol Exposures in the United States
Source: PLoS One. 2015 Nov 13;10(11):e0143044. doi: 10.1371/journal.pone.0143044 (PMC4643878; doi:10.1371/journal.pone.0143044)
Supplement: S3 Table — (PDF) [file pone.0143044.s007.pdf]

S3 Table. Years that States Required Addition of Denatonium Benzoate to Ethylene Glycol Based Antifreeze Formulations.

1991	Oregon
2002	California
2005	Arizona, Georgia, Illinois, Maine, Maryland, Massachusetts, New Jersey, New Mexico, Tennessee, Utah, Vermont, Virginia, Washington, West Virginia, Wisconsin
2006	Antifreeze Bittering Act introduced in 108th Congress but did not advance
2012	Consumer Specialty Products Association agreed to voluntarily add a bitter flavoring to antifreeze and engine coolant manufactured for sale in the consumer market in all 50 states and the District of Columbia
