# Supplementary material for: Clinical Features of Reported Ethylene Glycol Exposures in the United States
Source: PLoS One. 2015 Nov 13;10(11):e0143044. doi: 10.1371/journal.pone.0143044 (PMC4643878; doi:10.1371/journal.pone.0143044)
Supplement: S4 Table — (DOCX) [file pone.0143044.s008.docx]

**S4 Tables: Statistics from Logistic Regression Analysis of Risk Factors Associated with Ethylene Glycol Ingestion (Figure 1)**

**Figure 1A: Intentional Ingestion Model (all patients included, C-statistic =0.739)**

| Parameter |  | Estimate | P value | Odds  Estimate | 95% Wald Confidence Limits | |
| --- | --- | --- | --- | --- | --- | --- |
| Age | 19-29 vs <=18 | 1.4098 | <.0001 | 4.095 | 3.172 | 5.287 |
|  | 30-39 vs <=18 | 1.8836 | <.0001 | 6.577 | 5.044 | 8.576 |
|  | 40-49 vs <=18 | 1.8993 | <.0001 | 6.681 | 5.108 | 8.74 |
|  | 50-59 vs <=18 | 2.1704 | <.0001 | 8.761 | 6.618 | 11.599 |
|  | 60-64 vs <=18 | 2.0244 | <.0001 | 7.571 | 5.205 | 11.012 |
|  | 65-74 vs <=18 | 2.1043 | <.0001 | 8.201 | 5.446 | 12.351 |
|  | 75+ vs <=18 | 1.9185 | <.0001 | 6.811 | 3.802 | 12.201 |
| Gender | Female vs Male | 0.716 | <.0001 | 2.046 | 1.770 | 2.366 |
| Weight (kg) | 61-70 vs <=60 | 0.5452 | <.0001 | 1.725 | 1.357 | 2.193 |
|  | 71-80 vs <=60 | 0.1757 | 0.1545 | 1.192 | 0.936 | 1.518 |
|  | 81-90 vs <=60 | -0.0054 | 0.9667 | 0.995 | 0.773 | 1.28 |
|  | 91-100 vs <=60 | -0.2543 | 0.0546 | 0.775 | 0.598 | 1.005 |
|  | >100 vs <=60 | -0.2934 | 0.0269 | 0.746 | 0.575 | 0.967 |
| Oral Ingestion | 1 vs 0 | 2.4329 | <.0001 | 11.392 | 7.336 | 17.689 |
| Season | Spring vs Fall | 0.2828 | 0.0009 | 1.327 | 1.123 | 1.567 |
|  | Summer vs Fall | 0.1206 | 0.1637 | 1.128 | 0.952 | 1.337 |
|  | Winter vs Fall | -0.0267 | 0.7612 | 0.974 | 0.820 | 1.156 |

**Figure 1B: Death and Major Effect Model (all patients included, C-statistic=0.922)**

| Parameter |  | Estimate | P value | Odds | 95% Wald | |
| --- | --- | --- | --- | --- | --- | --- |
|  |  |  |  | Estimate | Confidence Limits | |
| Age | 19-29 vs <=18 | 0.465 | 0.0678 | 1.592 | 0.967 | 2.622 |
|  | 30-39 vs <=18 | 0.6591 | 0.0115 | 1.933 | 1.159 | 3.223 |
|  | 40-49 vs <=18 | 1.236 | <.0001 | 3.442 | 2.094 | 5.659 |
|  | 50-59 vs <=18 | 1.2625 | <.0001 | 3.534 | 2.116 | 5.902 |
|  | 60-64 vs <=18 | 1.5055 | <.0001 | 4.506 | 2.343 | 8.666 |
|  | 65-74 vs <=18 | 1.4961 | <.0001 | 4.464 | 2.214 | 9.003 |
|  | 75+ vs <=18 | 1.8662 | 0.0001 | 6.464 | 2.518 | 16.594 |
| Gender | Female vs Male | -0.0743 | 0.5916 | 0.928 | 0.708 | 1.218 |
| Weight (kg) | 61-70 vs <=60 | -0.1809 | 0.374 | 0.835 | 0.56 | 1.243 |
|  | 71-80 vs <=60 | -0.2298 | 0.2624 | 0.795 | 0.532 | 1.188 |
|  | 81-90 vs <=60 | -0.0791 | 0.7088 | 0.924 | 0.610 | 1.400 |
|  | 91-100 vs <=60 | -0.3592 | 0.1144 | 0.698 | 0.447 | 1.091 |
|  | >100 vs <=60 | -0.6017 | 0.0108 | 0.548 | 0.345 | 0.87 |
| Oral Ingestion | 1 vs 0 | 0.1323 | 0.7188 | 1.141 | 0.556 | 2.345 |
| Season | Spring vs Fall | 0.4301 | 0.0094 | 1.537 | 1.111 | 2.127 |
|  | Summer vs Fall | 0.3443 | 0.0432 | 1.411 | 1.011 | 1.970 |
|  | Winter vs Fall | 0.3996 | 0.0207 | 1.491 | 1.063 | 2.092 |
| Intentional Ingestion | 1 vs 0 | 3.957 | <.0001 | 52.299 | 37.216 | 73.496 |
