# Supplementary material for: Clinical Features of Reported Ethylene Glycol Exposures in the United States
Source: PLoS One. 2015 Nov 13;10(11):e0143044. doi: 10.1371/journal.pone.0143044 (PMC4643878; doi:10.1371/journal.pone.0143044)
Supplement: S5 Table — (DOCX) [file pone.0143044.s009.docx]

**S5 Table: Reported Reason of Exposure to Ethylene Glycol**

| **Report reason of exposure** | **Number of exposures (%)** | **Percent of total exposures (%)** |
| --- | --- | --- |
| **Adverse reaction all** | **154** | **<1** |
| Adverse reaction: drug | 5 (3) | <1 |
| Adverse reaction: other | 148 (96) | <1 |
| **Intentional all** | **7070** | **15** |
| Intentional: abuse | 226 (3) | <1 |
| Intentional: misuse | 861 (12) | 2 |
| Intentional: suicide attempt | 5606 (79) | 12 |
| Intentional: unknown | 377 (5) | <1 |
| **Other all** | **823** | **2** |
| Other: contamination/tampering | 257 (31) | <1 |
| Other: malicious | 564 (68) | 1 |
| **Unintentional all** | **38027** | **81** |
| Unintentional: bite/sting | 1 (<1) | <1 |
| Unintentional: environmental | 3582 (9) | 8 |
| Unintentional: food poisoning | 6 (<1) | <1 |
| Unintentional: misuse | 10112 (27) | 22 |
| Unintentional: general | 20917 (55) | 44 |
| Unintentional: occupational | 3123 (8) | 7 |
| Unintentional: therapeutic error | 37 (<1) | <1 |
| Unintentional: unknown | 249 (<1) | <1 |
| **Unknown reason** | **736** | **2** |
| **Total** | **46810** | **100** |
